# Regulation of temozolomide resistance in glioma cells via the RIP2/NF‐κB/MGMT pathway

**DOI:** 10.1111/cns.13591

**Published:** 2021-01-18

**Authors:** Yu‐Hua Hu, Bao‐Hua Jiao, Cheng‐Ye Wang, Jian‐Liang Wu

**Affiliations:** ^1^ Department of Neurosurgery The Second Hospital of Hebei Medical University Shijiazhuang China

**Keywords:** glioma, MGMT, NF‐κB, RIP2, temozolomide

## Abstract

**Background:**

Temozolomide (TMZ) is a first‐line chemotherapy drug for the treatment of malignant glioma and resistance to it poses a major challenge. Receptor‐interacting protein 2 (RIP2) is associated with the malignant character of cancer cells. However, it remains unclear whether RIP2 is involved in TMZ resistance in glioma.

**Methods:**

RIP2 expression was inhibited in TMZ‐resistant glioma cells and normal glioma cells by using small interfering RNA (siRNA) against RIP2. Plasmid transfection method was used to overexpress RIP2. Cell counting kit‐8 assays were performed to evaluate cell viability. Western blotting or immunofluorescence was performed to determine RIP2, NF‐κB, and MGMT expression in cells. Flow cytometry was used to investigate cell apoptosis. TMZ‐resistant glioma xenograft models were established to evaluate the role of the RIP2/NF‐κB/MGMT signaling pathway in drug resistance.

**Results:**

We observed that RIP2 expression was upregulated in TMZ‐resistant glioma cells, whereas silencing of RIP2 expression enhanced cellular sensitivity to TMZ. Similarly, upon the induction of RIP2 overexpression, glioma cells developed resistance to TMZ. The molecular mechanism underlying the process indicated that RIP2 can activate the NF‐κB signaling pathway and upregulate the expression of O6‐methylguanine‐DNA methyltransferase (MGMT), following which the glioma cells develop drug resistance. In the TMZ‐resistant glioma xenograft model, treatment with JSH‐23 (an NF‐κB inhibitor) and lomeguatrib (an MGMT inhibitor) could enhance the sensitivity of the transplanted tumor to TMZ.

**Conclusion:**

We report that the RIP2/NF‐κB/MGMT signaling pathway is involved in the regulation of TMZ resistance. Interference with NF‐κB or MGMT activity could constitute a novel strategy for the treatment of RIP2‐positive TMZ‐resistant glioma.

## Graphical abstract

Temozolomide is a first‐line chemotherapy drug for the treatment of malignant glioma and resistance to it poses a major challenge. An increasing number of studies report that RIP2 is associated with the malignant character of cancer cells. In this study, we report that the RIP2/NF‐κB/MGMT signaling pathway is involved in the regulation of TMZ resistance. Interference with NF‐κB or MGMT activity could constitute a novel strategy for the treatment of RIP2‐positive TMZ‐resistant glioma.

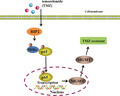

## 1 INTRODUCTION

Gliomas are common tumors of the central nervous system that pose a major threat to the physical and mental health of patients.^1^ As gliomas are formed in the brain, they are difficult to operate on and are mostly inaccessible to drugs that cannot cross the blood‐brain barrier. Therefore, clinical treatment of gliomas can be challenging, particularly in cases of high‐grade gliomas that undergo rapid progression and have poor prognosis.^1^, ^2^, ^3^ Currently, surgical treatment, radiotherapy, and chemotherapy are the major treatment methods for malignant gliomas. Novel therapies, such as molecular targeted therapy and immunotherapy, have also yielded positive clinical outcomes.^4^, ^5^ However, the majority of gliomas are invasive and difficult to treat, primarily because the boundary between the tumors and the surrounding brain tissue is unclear.^6^

Alkylating agents are important antitumor drugs used in clinical practice.^7^, ^8^ Among them, those that induce the formation of O6‐methylguanine pose a major threat to cells and can induce mutation and death. These agents can cross the blood‐brain barrier and are nearly 100% bioavailable.^9^ Temozolomide (TMZ) significantly improves the prognosis of patients with malignant glioma. Compared with traditional chemotherapy drugs, TMZ causes fewer adverse reactions and is the best first‐line drug for glioma treatment.^10^, ^11^, ^12^ However, ever since TMZ has been used in clinical practice, there have been multiple reports stating that the rate of clinical efficacy of TMZ is less than 45%. Few of the patients treated with TMZ reported that though the short‐term effects were encouraging, the long‐term effects were not ideal.^13^, ^14^, ^15^ This is because tumor cells develop primary or secondary resistance to TMZ. Resistance of glioma cells to TMZ is affected by several factors, such as tumor stem cells and their microenvironment, the stress response of tumor cells to chemotherapy drugs, the permeability of drugs in tumor tissues, and DNA damage and repair.^16^, ^17^, ^18^, ^19^ Receptor‐interacting protein 2 (RIP2) belongs to the RIP family of proteins and is expressed in various tissues. RIP2 interacts with a variety of proteins, participates in multi‐channel signal transduction, and executes the associated physiological functions. It is considered to form an important link between innate immunity, adaptive immunity, and inflammation.^20^ RIP2 was shown to activate NF‐κB, promoting anti‐taxol‐ and ceramide‐induced apoptosis in TNBC cells.^21^ RIP2 was also suggested to be involved in drug resistance in triple‐negative breast cancer. Dong et al^22^ reported that the paired box protein 5 (Pax5) interacts with RIP2 to promote NF‐κB activation and drug resistance in B‐lymphoproliferative disorders. In our previous study, we showed that RIP2 expression increased in gliomas; additionally, findings from in vitro studies indicated that RIP2 could activate the NF‐κB and p38 signaling pathways and subsequently influence the biological behavior of malignant gliomas.^23^ However, it remained unclear whether RIP2 is involved in TMZ resistance in glioma. In this paper, we report that RIP2 expression is upregulated in TMZ‐resistant neuroglioma cells and that RIP2 silencing enhances cellular sensitivity to TMZ. The biological effects of RIP2 are mediated through activation of NF‐κB for upregulation of MGMT expression. In the TMZ‐resistant glioma cell xenograft model, inhibition of NF‐κB and MGMT enhances the response of the transplanted tumor to TMZ. These findings help determine the mechanism of RIP2 resistance to TMZ in gliomas. NF‐κB and MGMT may serve as valuable therapeutic targets in RIP2‐positive gliomas.

## 2 MATERIALS AND METHODS

### 2.1 Reagents

Temozolomide (34219) was obtained from Sigma‐Aldrich. SC75741 (HY‐10496), SN50 (HY‐P0151), JSH‐23 (HY‐13982), and lomeguatrib (HY‐13668) were obtained from MedChemExpress. Primary antibodies against NF‐κB p65 (D14E12) (59674S, 1:1000), p‐NF‐κB p65 (Ser536) (93H1) (3033S, 1:1000), and IκBα (44D4) (4812, 1:1000) were purchased from Cell Signaling Technology. Primary antibodies against MGMT (ab69629) were purchased from Abcam. Primary antibodies against Lamin B1 (B‐10) (sc‐374015) were purchased from Santa Cruz Biotechnology. Primary antibodies against GAPDH (1A6) (MB001, 1:1000) were purchased from Bio‐World. Anti‐mouse IgG, HRP‐linked antibody, and anti‐rabbit IgG, HRP‐linked antibodies were purchased from Santa Cruz Biotechnology. Matrigel (354248) was obtained from BD Biosciences.

### 2.2 Cell cultures

**FIGURE 1:**
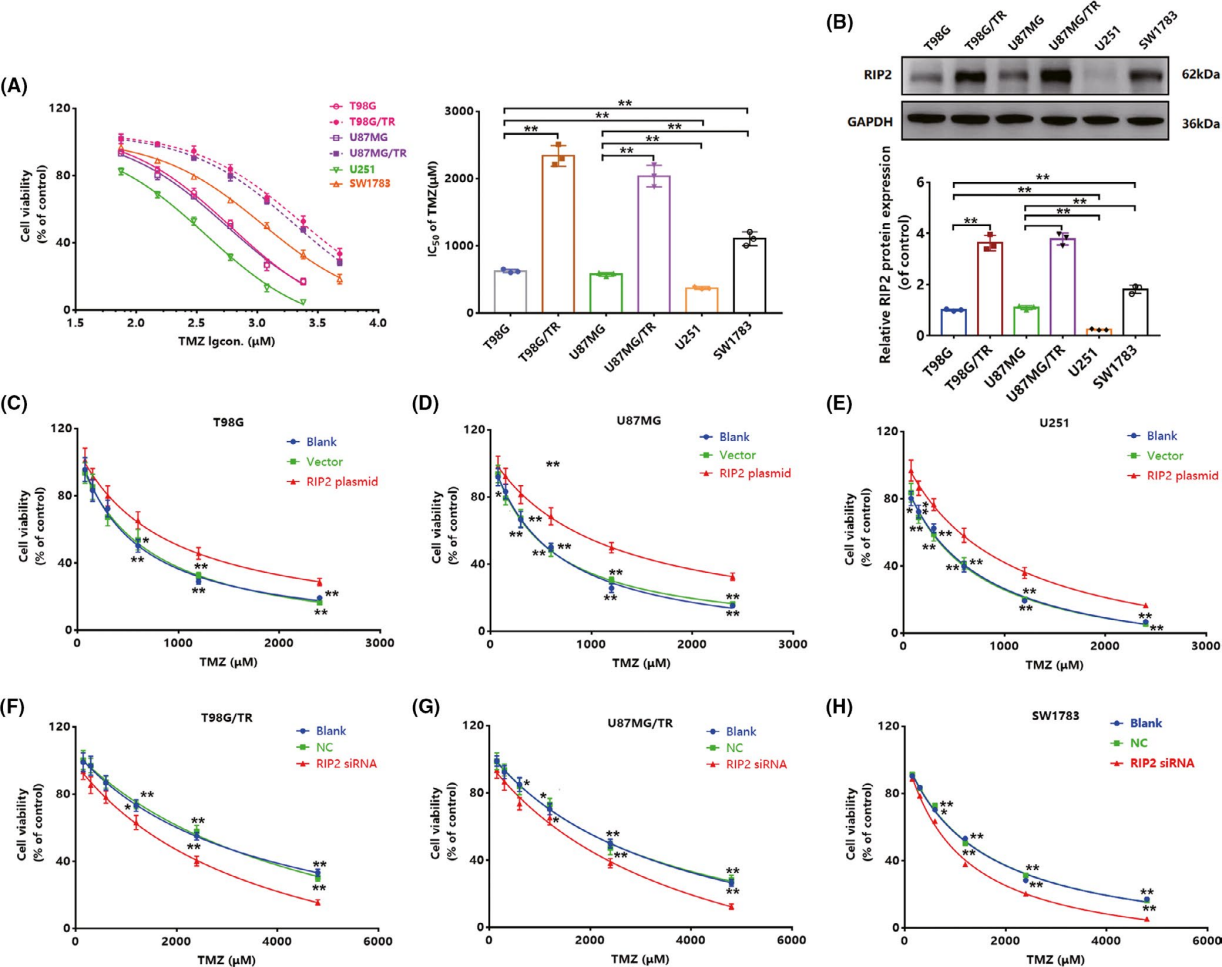
Receptor‐interacting protein 2 (RIP2) decreased the sensitivity of glioma cells to TMZ. (A) Viability of glioma cells was determined using the CCK‐8 method 72 hours after treatment with TMZ (75, 150, 300, 600, 1200, and 2400 μM), and the IC50 value was calculated according to the results of the CCK‐8 assay. (B) RIP2 expression was observed using Western blotting. The results were normalized to GAPDH expression and expressed in terms of fold change in comparison with expression in T98G cells. (C‐E) T98G, U87MG, and U251 cells were transfected with RIP2 plasmid and empty vector, and the activity of each cell was determined after 72 hours of treatment with TMZ (75, 150, 300, 600, 1200, and 2400 μM). (F‐H) T98G/TR, U87MG/TR, and SW1783 cells were transfected with RIP2 siRNA and NC siRNA, and the activity of each cell was determined after 72 hours of treatment with TMZ (150, 300, 600, 1200, 2400, and 4800 μM). Results in (A)–(H) are expressed in terms of mean ± SD from three independent experiments

T98G, U87MG, and SW1783 glioma cell lines were purchased from the American Type Culture Collection (ATCC). The U251 glioma cell line was purchased from the Culture Collection of the Chinese Academy of Science. The medium for culturing SW1783 cells was RPMI 1640 (Invitrogen). The medium for culturing T98G, U87MG, and U251 cells was DMEM (high glucose) (Invitrogen). The four glioma cell lines were maintained in a 5% CO_2_ atmosphere at 37°C in medium supplemented with 100 U/ml penicillin, 100 µg/ml streptomycin (HyClone), and 10% FBS. The resistant variants of the T98G/TR cells were developed by culturing initial T98G cells with increasing concentrations of TMZ (from 75 to 4800 μM). For U87MG/TR cells, the resistant variants were developed by culturing initial U87MG cells with increasing concentrations of TMZ (from 75 to 2400 μM). TMZ was added every 48 hours. Cell viability was analyzed every month using the CCK‐8 assay. IC50 values of T98G, U87MG, T98G/TR, and U87MG/TR are presented in Figure 1A. TMZ was added at a final concentration of 1 mM to maintain the resistant phenotypes till 1 week prior to the experiments.

### 2.3 Cell viability assay

Cell viability was evaluated using the CCK‐8 (Cell Counting Kit‐8; Solarbio) assay. The cell suspension (100 μl/well) was inoculated in a 96‐well plate, and the plate was pre‐incubated in a humidified incubator (37°C, 5% CO_2_). The cells were treated for 72 hours. CCK‐8 solution (10 μl) was added to each well of the plate. The plate was incubated for 4 hours in the incubator. Absorbance was measured at 450 nm using an ELx800 plate reader (BioTek).

### 2.4 Transfection of RIP2 plasmid

The plasmid pEX‐3 containing the RIP2 gene (NM_003821.1) was constructed by GenePharma, Inc., and transfection was performed using GenJet™ Plus DNA In vitro Transfection Reagent (Signagen, SL100499) in accordance with the manufacturer's protocol.

### 2.5 siRNA interference

To inhibit RIP2 expression, small interfering RNA (siRNA) against RIP2 was used. RIP2‐targeting siRNA was designed and manufactured by GenePharma, Inc. The siRNA was added to 5 μl of RNase‐free water at a concentration of 100 pmol/μl, mixed with 10 μl of the transfection reagent, and maintained at 25°C for 15 minutes. For the transfection assay, 5 × 10^6^ cells were seeded in 6‐well plates and incubated overnight at 37°C in 5% CO_2_ until 80%‐90% confluence was achieved and were then transfected with siRNA.

### 2.6 Western blotting

Cells were lysed with RIPA lysate to obtain total protein. Total proteins were separated by electrophoresis on 15% SDS‐PAGE and then transferred onto a PVDF membrane of 0.2 µm pore size (Millipore) for Western blot analysis. The PVDF membrane was blocked with 5% skim milk powder in Tris‐buffered saline/1% Tween‐20 (TBS‐T) for 2 hours at 4°C and then incubated with the primary antibodies anti‐RIP2 (monoclonal, rabbit anti‐human, 1:1000), anti‐NF‐κB p65 (monoclonal, rabbit anti‐human, 1:2000), anti‐p‐NF‐κB p65 (monoclonal, rabbit anti‐human, 1:8000), anti‐IκBα (monoclonal, mouse anti‐human, 1:1000), anti‐MGMT (monoclonal, rabbit anti‐human, 1:1500), anti‐LaminB (monoclonal, rabbit anti‐human, 1:1000), and anti‐GAPDH (monoclonal, rabbit anti‐human, 1:1000) (Cell Signaling Technology) overnight at 4°C. Afterward, the membranes were washed thrice with TBS‐T for 10 minutes and were incubated with horseradish peroxidase (HRP)‐labeled secondary antibodies of sheep to mouse and sheep to rabbit (ZSGB‐BIO Co., Ltd.) at 4°C for 1 hour. The non‐binding secondary antibodies were washed out thrice by TBS‐T for 10 minutes. Protein bands were detected using a SuperSignal West Femto Substrate (Thermo Fisher) and detected using a GE AI600 system (GE). Densitometric analysis of protein bands was performed using ImageQuant TL7.0 (GE).

### 2.7 Cell apoptosis analysis by flow cytometry

A total of 5 × 10^5^ cells were collected and fixed in 70% ice cold ethanol overnight. Cells were then incubated with 10 mg/ml RNase (Sigma) and 50 mg/ml propidium iodide (Sigma) at 37°C for 30 minutes in dark. The treated cells were harvested and incubated with reagents from the Annexin V‐FITC apoptosis kit (BioVision) according to the manufacturer's protocol. Cell apoptosis was analyzed by flow cytometry (BD Bioscience).

### 2.8 Immunofluorescence analysis

Cells were plated on coverslips and then fixed with pre‐cooled methanol on ice for 20 minutes. Cells were then washed with PBST (PBS, 0.1% Triton X‐100), followed by blocking with 3% BSA at 15 ~ 25℃ for 30 minutes. Cells were incubated with primary antibodies for MGMT and then diluted in 3% BSA at 1:100 for 2 hours at room temperature. After washing thrice with PBST, the cells were incubated with secondary antibodies in 3% BSA for 2 hours. Nuclei were stained with 0.5 μg/ml of DAPI (PBST) at room temperature for 10 minutes. Cells were mounted with ProLong^®^ Gold Antifade (P36930, Thermo Fisher) and viewed with a confocal system under an FV1000 inverted microscope (Olympus). Images were analyzed using the scientific software module of Imaris.

### 2.9 Animal experiments

Animal experiments were conducted in accordance with the Declaration of Helsinki and the Regulations for Care and Use of Laboratory Animals of the State Food and Drug Administration of China. The experimental protocols were approved by the Animal Ethics Committee of the Second Hospital of Hebei Medical University. Male nude mice (BALB/c‐nu) were obtained from GemPharmatech Co., Ltd. [License # SCXK(SU) 2018–0008] and maintained in a 12‐hour day/night cycle with free access to food and water. The glioma cells T98G/TR and U87MG/TR were resuspended in DMEM at 6 × 10^6^ cells per 200 µl and were injected subcutaneously into nude mice. Tumor diameter in the nude mice was measured daily using digital calipers. After the tumor volume reached 100 mm^3^, the mice were randomly divided into six groups (*n* = 6 mice per group): solvent control group, lomeguatrib group, JSH‐23 group, TMZ group, TMZ + lomeguatrib group, and TMZ + JSH‐23 group. TMZ (20 mg/kg i.p.), lomeguatrib (8 mg/kg i.p.), and JSH‐23 (3 mg/kg p.o.) were administered once a day for three weeks. The solvent control group was administered an equal volume of saline. Tumor sizes were measured thrice per week, and the volume was calculated using the formula: tumor volume (mm^3^)  = (length × width^2^) × 1/2. The animals were anesthetized and sacrificed on day 21 after inoculation. Tumors were collected, weighed, and imaged and fixed with 10% neutral buffered formalin for pathological confirmation.

### 2.10 MGMT activity assay

MGMT activity was quantified using the MGMT Activity Assay Kit (BioVision, Inc.) according to the manufacturer's instructions. Briefly, 2 × 10^6^ cells were resuspended in 100 μl extract buffer, put on ice for 20 minutes, and centrifuged at 12,000 *g* for 10 minutes at 4°C. The supernatants were collected and diluted 10 times with ddH_2_O, and the protein concentration was measured. The supernatants (containing ~50–200 μg of proteins) were diluted to 85 μl with extraction buffer. Positive and negative control wells were set up, and 10 μl of 10× reaction buffer and 5 μl calpain substrate were added into each well. After incubation in dark at 37°C for 1 hour, the fluorescence intensity of the samples was measured using a plate reader (BioTech) with excitation at 400 nm and emission at 505 nm.

### 2.11 Histological analysis

Xenograft tumors were fixed with a 4% formaldehyde solution in PBS, embedded in paraffin, and sectioned. Following deparaffinization with xylene and hydration with decreasing concentrations of alcohol, the sections were incubated with 0.3% hydrogen peroxide to block endogenous peroxidase activity and boiled in EDTA buffer (pH = 8.0) for antigen retrieval. Sections were then incubated overnight with mouse monoclonal MGMT antibody at 4°C in a moist chamber. On the next day, after washing with PBS, the samples were incubated with HRP‐conjugated secondary antibody (ZSGB‐BIO Co., Ltd.) before microscopy analysis. The integrated optical density (IOD) values of tissue sections in each group were measured by Image‐Pro Plus 6.0 software (Media Cybernetics, Inc.).

### 2.12 Data and statistical analysis

All experiments were performed independently at least three times, and the data were analyzed using SPSS 19.0 and GraphPad Prism 7.0 for Windows. All data conform to the normal distribution by Shapiro‐Wilk test. All the results are expressed in terms of mean ± standard deviation (SD). Statistical significance was calculated using one‐way analysis of variance (ANOVA), followed by Fisher's multiple comparison test. *p* value <0.05 indicated statistical significance.

## 3 RESULTS

### 3.1 RIP2 plays a role in glioma cell resistance to TMZ

To explore the biological role of RIP2 in glioma cells, we first evaluated the viability of six types of glioma cells (T98G, T98G/TR, U87MG, U87MG/TR, U251, and SW1783) upon treatment with TMZ at different concentrations. Following TMZ treatment, T98G/TR and U87MG/TR cells had the highest viabilities and the highest TMZ inhibitory concentrations (IC50), followed by SW1783, whereas T98G, U87MG, and U251 cells had low viabilities and TMZ IC50 values, of which U251 cells were the least viable and had the lowest TMZ IC50 value (Figure 1A). Concurrently, we evaluated the differences in RIP2 expression in the six types of glioma cells. In each cell type, RIP2 expression shared positive correlation with cell viability and IC50 values (Figure 1B), suggesting an association between RIP2 expression and the effect of TMZ treatment in glioma cells.

To further confirm the role of RIP2 in TMZ chemoresistance, we induced RIP2 overexpression by transfecting the three types of glioma cells having low RIP2 expression with a RIP2 plasmid. Compared to that in the vector, the three types of cells transfected with RIP2 exhibited better viability upon TMZ treatment (Figure 1C‐G). Moreover, we used siRNA technology to hinder the expression of RIP2 in T98G/TR, U87MG/TR, and SW1783 cells. After RIP2 was disturbed, the viability of the three cells was significantly reduced (Figure 1F‐H). These results indicate that RIP2 is closely associated with resistance to TMZ in glioma cells.

### 3.2 RIP2 reduces the sensitivity of glioma cells to TMZ by regulating the NF‐κB pathway

**FIGURE 2:**
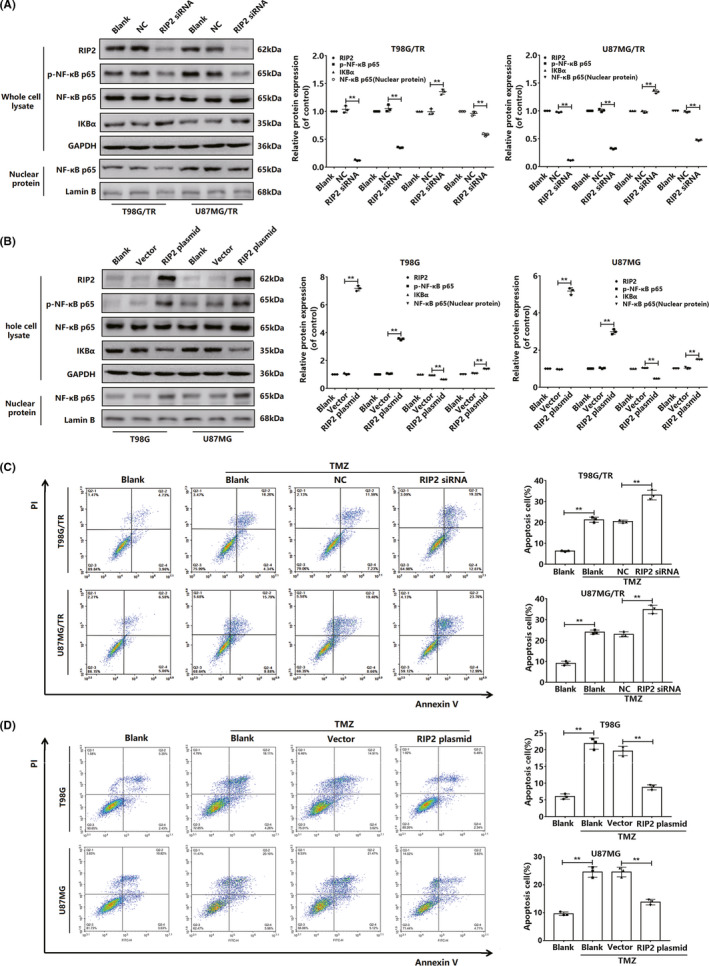
RIP2 reduces the sensitivity of glioma cells to TMZ by regulating the NF‐κB pathway. (A) Representative Western blot bands of p‐NF‐κB p65, NF‐κB p65, IκBα, and nuclear NF‐κB p65 extracted from T98G/TR and U87MG/TR cells transfected with RIP2 siRNA or NC siRNA. The results were normalized to GAPDH (LaminB) expression and expressed as the fold change in comparison with expression in the blank. (B) Expression of p‐NF‐κB p65, NF‐κB p65, IκBα, and nuclear NF‐κB p65 protein was observed in T98G/TR and U87MG/TR cells transfected with RIP2 plasmid and empty vector. The results were normalized to GAPDH (LaminB) expression and expressed as the fold change in comparison with expression in the blank. (C) T98G/TR and U87MG/TR cells transfected with RIP2 siRNA or NC siRNA were treated with TMZ (2400 μM) for 24 hours, and next, apoptosis was measured by flow cytometry. (D) T98G and U87MG cells transfected with RIP2 plasmid or empty vector were treated with TMZ (600 μM) for 24 hours, and next, apoptosis was measured by flow cytometry. Results in (A), (B), (C), and (D) are expressed in terms of mean ± SD from three independent experiments. **p < 0.01

Next, we silenced RIP2 expression in T98G/TR and U87MG/TR cells and subsequently, observed the sensitivity of the cells to TMZ to elucidate the underlying signaling mechanism. First, T98G/TR and U87MG/TR cells were treated with TMZ, stained with Annexin V‐FITC/PI, and observed using flow cytometry. Results indicated that after RIP2 silencing, total apoptosis of the two cell types increased significantly (Figure 2C). This implied that RIP2 silencing led to reduction in TMZ resistance in the two TMZ‐resistant cell lines. To further confirm the signaling mechanism, we detected NF‐κB signaling proteins using Western blotting. Analysis of the expression levels of NF‐κB p65, p‐NF‐κB p65, and IκBα in total cell proteins revealed that while RIP2 silencing did not affect the expression of total NF‐κB p65, it induced downregulation of p‐NF‐κB p65 expression and upregulation of IκBα expression (Figure 2A).

To confirm the regulatory mechanism of RIP2, we simultaneously monitored the response of RIP2‐overexpressing T98G and U87MG cells to TMZ treatment. Results of flow cytometry experiments indicated that RIP2 overexpression led to reduction in the total apoptosis rate of both cells (Figure 2D). RIP2 overexpression also led to reduced sensitivity to TMZ in T98G and U87MG cells. We observed that while RIP2 overexpression did not affect total NF‐κB p65 expression, it induced upregulation of p‐NF‐κB p65 expression and downregulation of IκBα expression in T98G and U87MG cells (Figure 2B). Analysis of protein expression in the nucleus revealed that NF‐κB p65 expression was significantly upregulated after RIP2 overexpression (Figure 2B). To conclude, RIP2 activates the NF‐κB signal pathway and reduces TMZ sensitivity in glioma cells.

### 3.3 RIP2 induces upregulation of MGMT expression in glioma cells

**FIGURE 3:**
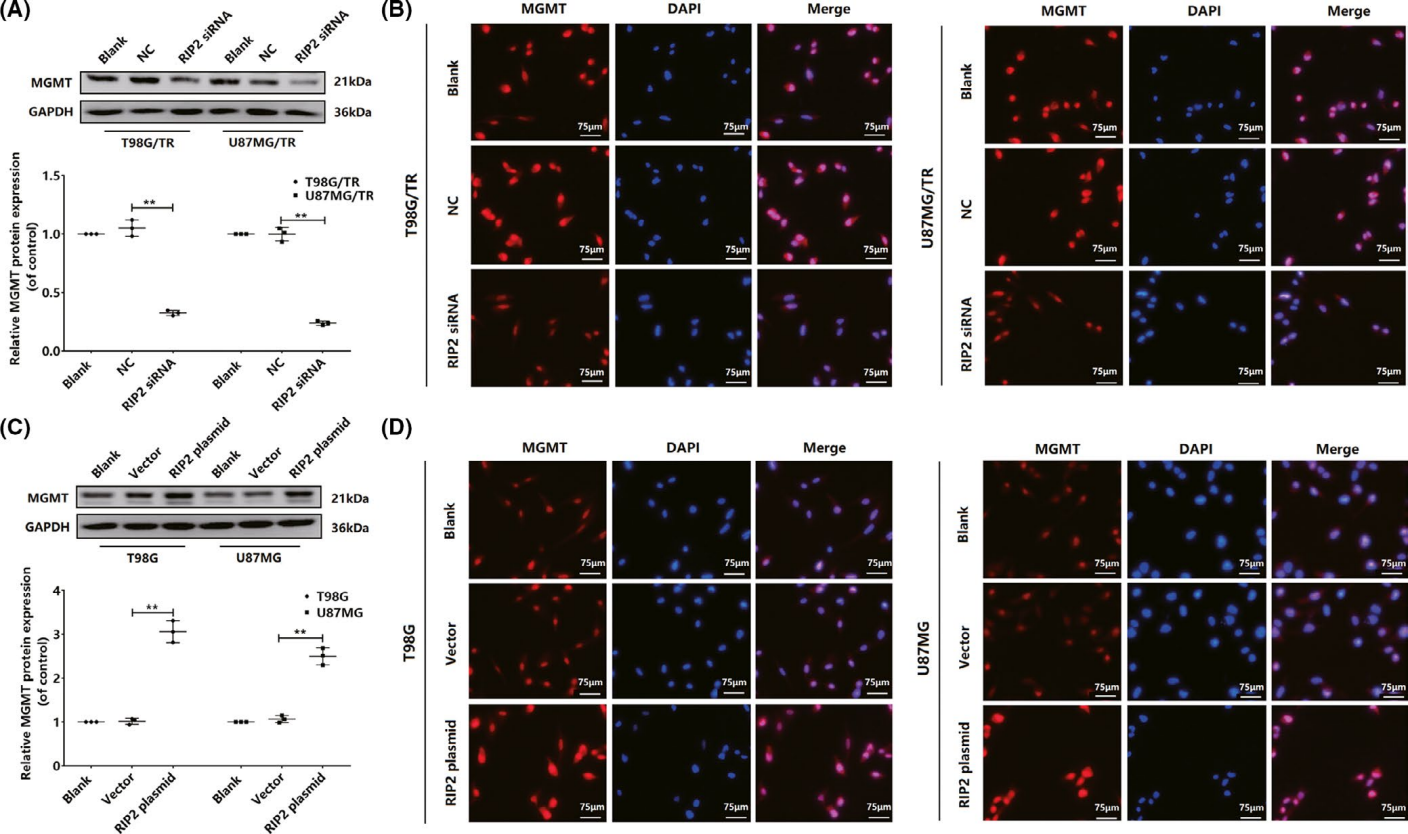
RIP2 induced upregulation of MGMT expression in glioma cells. (A) Relative protein expression levels of MGMT in T98G/TR and U87MG/TR cells transfected with RIP2 siRNA or NC siRNA. The results were normalized to GAPDH expression and expressed in terms of the fold change in comparison with expression in the blank. (B) Immunofluorescence of MGMT in T98G/TR and U87MG/TR cells transfected with RIP2 siRNA or NC siRNA (400×). Scale bars: 75 μm. (C) Relative protein expression levels of MGMT in T98G and U87MG cells transfected with RIP2 and empty vector. The results were normalized to GAPDH expression and presented in terms of the fold change in comparison to expression in the blank. (D) Immunofluorescence of MGMT in T98G and U87MG cells transfected with RIP2 plasmid and empty vector (400×). Scale bars: 75 μm. Results in (A) and (C) are expressed in terms of mean ± SD from three independent experiments. **p < 0.01

O6‐methylguanine‐DNA methyltransferase (MGMT) is a DNA repair protein that repairs the damage inflicted by alkylating agents (such as TMZ) upon tumor cell DNA and thus imparts resistance to alkylating agents in tumor cells. Herein, we focused on the alterations in MGMT protein expression. MGMT expression was significantly downregulated in T98G/TR and U87MG/TR cells upon RIP2 silencing (Figure 3A), whereas it was significantly upregulated in T98G and U87MG cells upon RIP2 overexpression (Figure 3C). We further examined MGMT expression pattern in immunofluorescence experiments. The results were similar to those of Western blotting experiments (Figure 3B,D). RIP2 was observed to upregulate MGMT expression in glioma cells.

### 3.4 Changes in MGMT expression induced by RIP2 in glioma cells are mediated through NF‐κB

**FIGURE 4:**
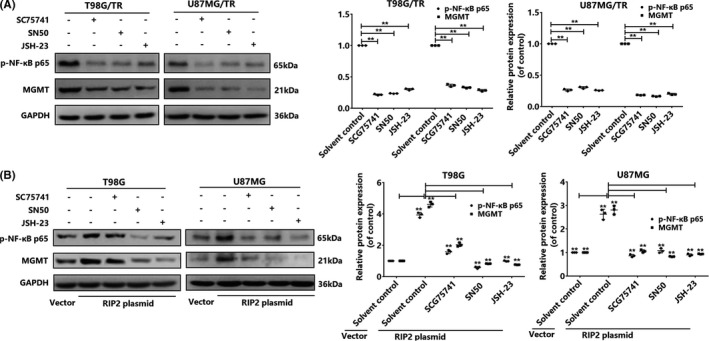
NF‐κB mediates the change in MGMT expression induced by RIP2 in glioma cells. (A) NF‐κB inhibitors SC75741 (1 μM), SN50 (18 μM), and JSH‐23 (20 μM) inhibit MGMT protein expression in T98G/TR and U87MG/TR cells. The results were normalized to GAPDH expression and presented as the fold change in comparison with expression in the solvent control. (B) NF‐κB inhibitors SC75741, SN50, and JSH‐2 inhibit the expression of MGMT protein in T98G and U87MG cells that overexpress RIP2. The results were normalized to GAPDH expression and presented as the fold change in comparison with expression in the solvent control. Results in (A) and (B) are expressed in terms of mean ± SD from three independent experiments. **p < 0.01, ^##^ *p* < 0.01

Our study revealed that RIP2 induces the NF‐κB signaling pathway and reduces the sensitivity of glioma cells to TMZ. RIP2 was observed to induce upregulation of MGMT expression in glioma cells. Whether RIP2 regulated MGMT expression by activating the NF‐κB pathway will need to be determined in further studies. In our study, we first used chemical inhibitors of NF‐κB (SC75741, SN50, and JSH‐23) to pretreat T98G/TR and U87MG/TR cells. After 48 hours, we extracted the total protein and observed that MGMT expression was downregulated in both cells (Figure 4A). Next, we studied RIP2 overexpression in T98G and U87MG cells. Results revealed that the three types of NF‐κB inhibitors inhibited RIP2 overexpression by varying protein expression (Figure 4B). Accordingly, we confirmed that RIP2 induces MGMT expression in glioma cells through the NF‐κB pathway.

### 3.5 Inhibition of NF‐κB/MGMT increases TMZ resistance in glioma cells

**FIGURE 5:**
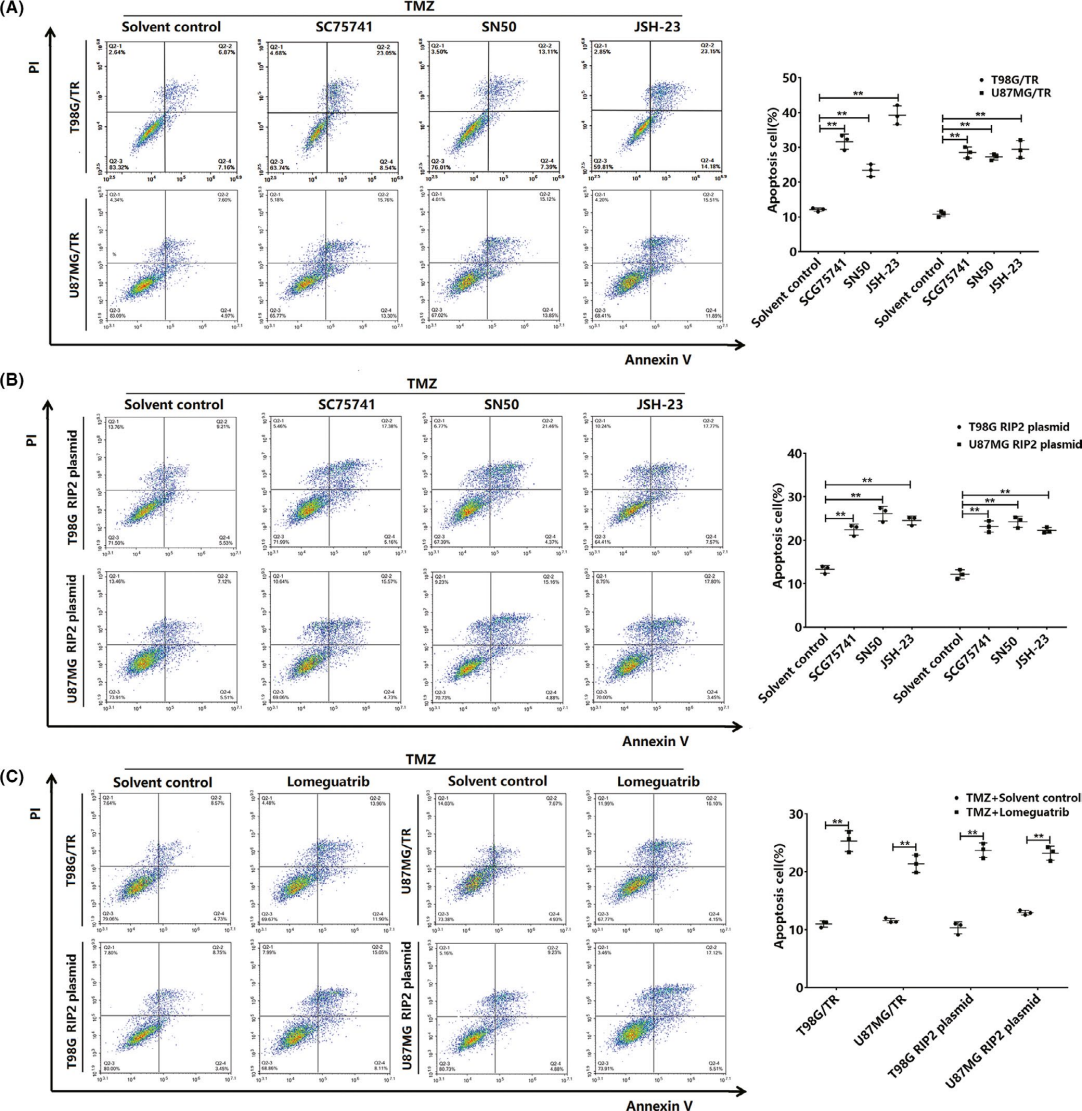
Inhibition of NF‐κB or MGMT enhances the sensitivity of glioma cells to TMZ. (A) T98G/TR and U87MG/TR cells were pretreated with the NF‐κB inhibitors SC75741, SN50, and JSH‐23 and then treated with TMZ for 24 hours. Apoptosis was measured by flow cytometry. (B) T98G and U87MG cells overexpressing RIP2 were pretreated with the NF‐κB inhibitors SC75741, SN50, and JSH‐23 and then treated with TMZ for 24 hours. Apoptosis was measured by flow cytometry. (C) T98G/TR and U87MG/TR cells and RIP2‐overexpressing T98G and U87MG cells treated with lomeguatrib for 24 hours. Apoptosis was measured by flow cytometry. Results in (A), (B), and (C) are expressed in terms of mean ± SD of three independent experiments. **p < 0.01

We confirmed that RIP2 can regulate MGMT expression in glioma cells through the NF‐κB pathway. Considering that RIP2 can induce TMZ resistance in glioma cells, we pretreated the resistant cells (T98G/TR and U87MG/TR) and RIP2‐overexpressing cells (T98G and U87MG) with NF‐κB chemical inhibitors (SC75741, SN50, and JSH‐23) and an MGMT chemical inhibitor (lomeguatrib), respectively. After 48 hours of treatment with TMZ, TMZ sensitivity of the pretreated cells increased significantly (Figure 5A‐C). The results indicated that inhibition of NF‐κB or MGMT could enhance the sensitivity of glioma cells to TMZ.

### 3.6 Inhibition of NF‐κB/MGMT can enhance TMZ sensitivity in the drug‐resistant glioma xenotransplantation model

**FIGURE 6:**
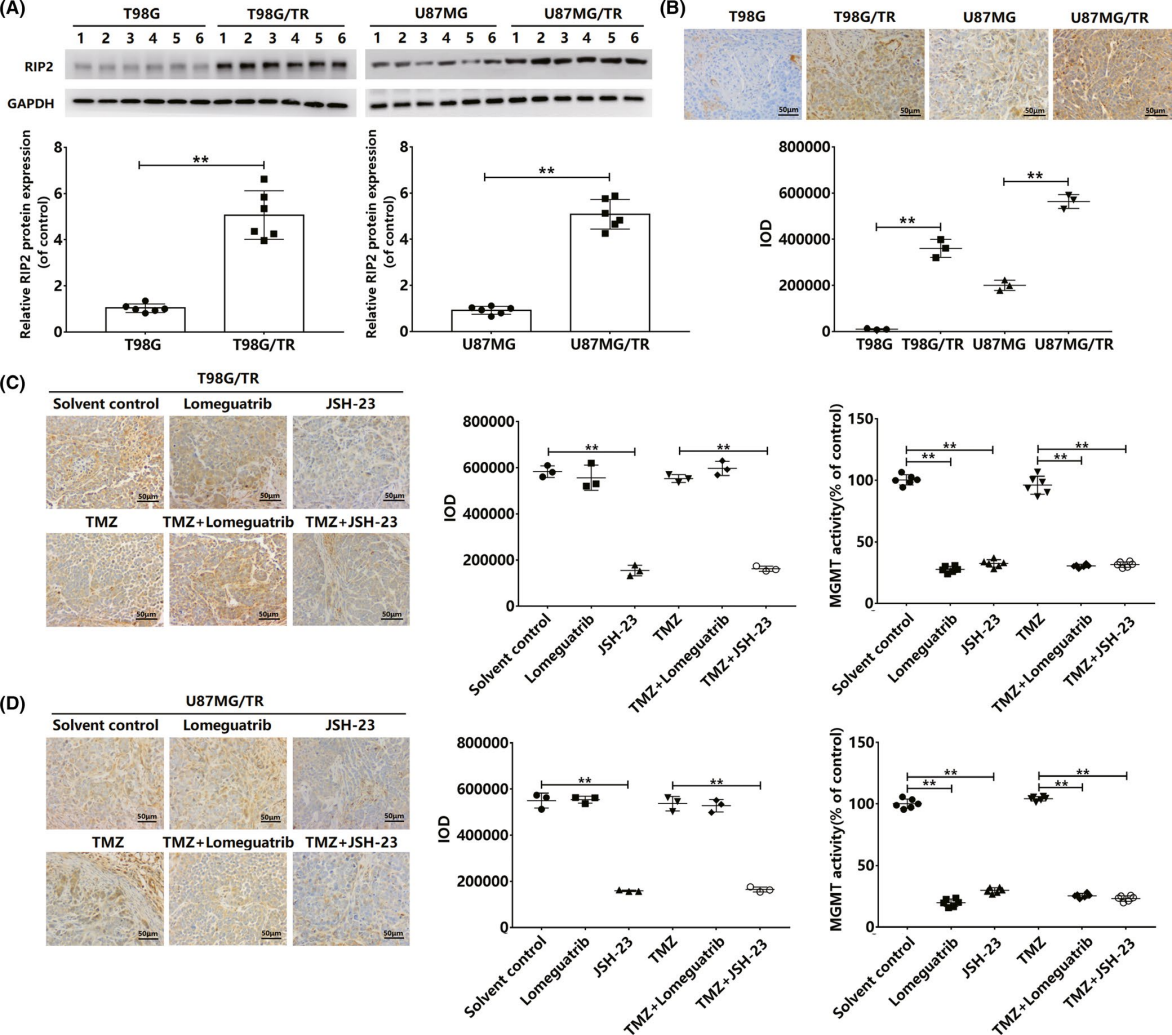
Histological results of T98G/TR and U87MG/TR xenografts at the end of the experiment. (A) Relative RIP2 protein expression levels in T98G/TR and U87MG/TR xenografts. The results were normalized to GAPDH expression and expressed in terms of fold change in comparison with expression in the T98G or U87MG. (B) Representative image (400×) of T98G,T98G/TR,U87MG,and U87MG/TR tumor sections stained with RIP2 antibody. Scale bar: 50 μm. (C) Representative image (400×) of the T98G/TR tumor section stained with MGMT antibody. Scale bar: 50 μm. Activity of MGMT in T98G/TR tumor tissue lysate. (D) Representative image (400×) of the U87MG/TR tumor section stained with MGMT antibody. Scale bar: 50 μm. Activity of MGMT in U87MG/TR tumor tissue lysate. Results in (A‐D) are expressed in terms of mean ± SD of three independent experiments. **p < 0.01

**FIGURE 7:**
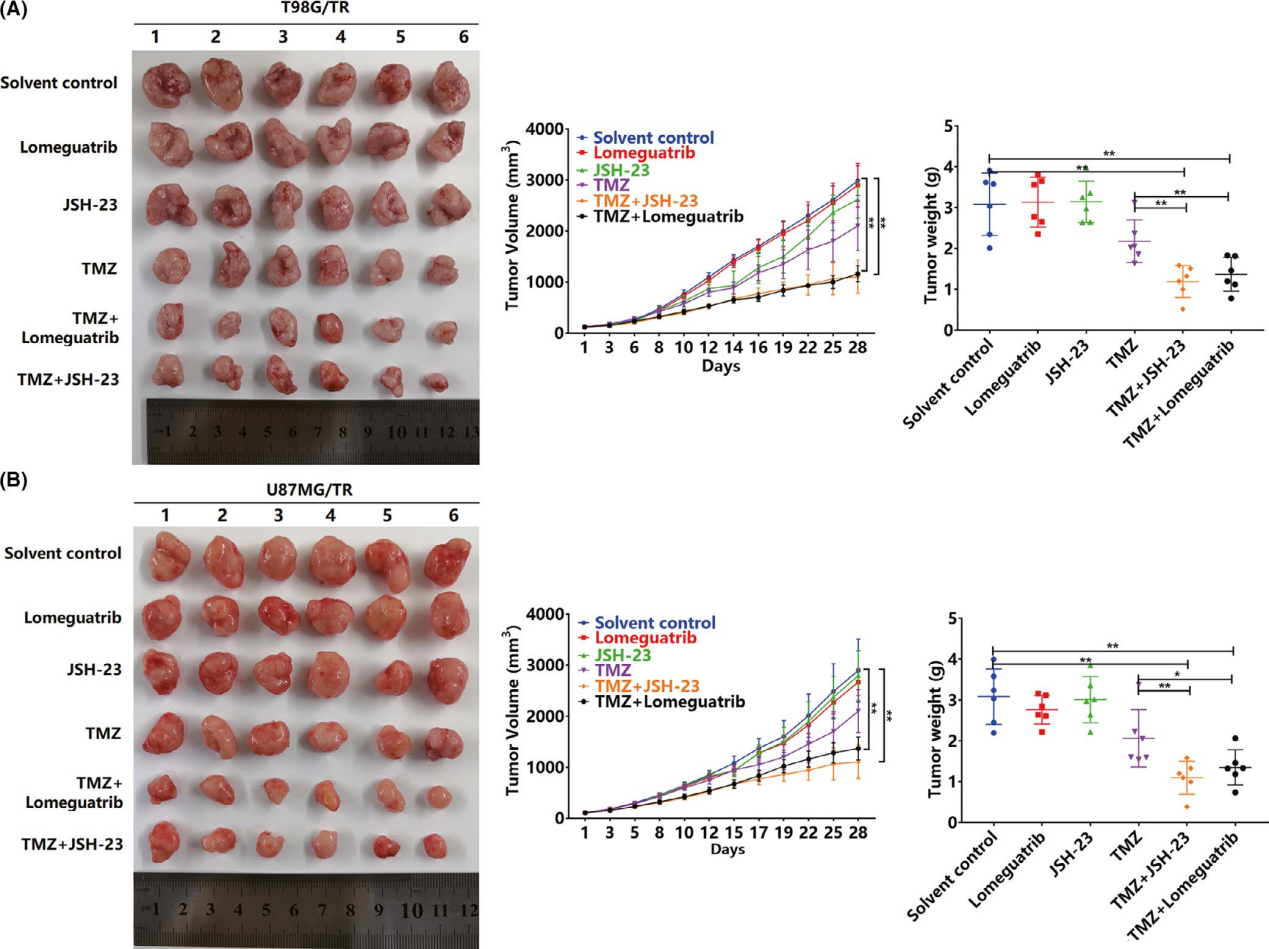
Inhibition of NF‐κB or MGMT enhances the sensitivity of T98G/TR and U87MG/TR xenografts to TMZ. (A) The qualified tumor‐bearing mice were divided into groups and treated with drugs for 28 days. Images of tumor tissue, tumor weight, and tumor volume of T98G/TR xenografts in nude mice. (B) The qualified tumor‐bearing mice were divided into groups and treated with drugs for 28 days. Images of tumor tissue, tumor weight, and tumor volume of U87MG/TR xenografts in nude mice. Results in (A‐B) are expressed in terms of mean ± SD. **p < 0.01

We compared the expression of RIP2 in normal glioma xenograft tissues (T98G and U87MG) and TMZ‐resistant glioma xenograft tissues (T98G/TR and U87MG/TR). Results showed that RIP2 was upregulated in T98G/TR and U87MG/TR xenograft tissues (Figure 6A,B). To confirm the role of RIP2 in TMZ resistance in gliomas, we constructed the T98G/TR and U87MG/TR xenograft models. Immunohistochemical analysis revealed that the number of MGMT‐positive in cells pretreated with JSH‐23 reduced significantly, whereas lomeguatrib pretreatment did not affect MGMT expression (Figure 6C,D). Additionally, MGMT activity was significantly lower in the two xenografts pretreated with JSH‐23 or lomeguatrib than in the control group (Figure 6C,D). In the two xenograft tumor models, JSH‐23 or lomeguatrib itself has no obvious effect on tumor growth, but both can enhance the therapeutic effect of TMZ; thus, tumor volume is significantly reduced, and tumor weight is significantly reduced (Figure 7A,B).

## 4 DISCUSSION

Temozolomide is a first‐line chemotherapeutic drug for the treatment of malignant glioma. Although the application of TMZ has led to improvements in the survival and quality of life of patients with malignant glioma, TMZ resistance remains a major contributing factor to the failure of malignant glioma treatment. RIP2, also known as RICK/CARDIAK, consists of an N‐terminal serine/threonine kinase domain and a CARD domain for protein‐protein interaction.^20^ RIP2 plays a physiological or pathological role in processes related to immunity and inflammation. Studies conducted in recent years have revealed that RIP2 is related to the proliferation, migration, invasion, and metastasis of malignant tumors.^23^, ^24^, ^25^ RIP2 has also been reported to activate NF‐κB to promote chemotherapy resistance in triple‐negative breast cancer cells.^21^ RIP2 interacts with PAX5 to promote NF‐κB activation and drug resistance in B‐lymphoproliferative disorders.^22^ However, there is no evidence to confirm the association between RIP2 and drug resistance in gliomas.

In this study, we first observed the expression of RIP2 in six glioma cell lines, including two TMZ‐resistant cell lines, and assessed the sensitivity of the cells to TMZ. We observed that RIP2 expression level was the highest in TMZ‐resistant cell lines and was closely related to TMZ resistance in the other four cell lines. Concurrently, similarly to that observed in earlier reports, upregulation of RIP2 expression led to activation of the NF‐κB signaling pathway.^21^, ^22^, ^23^ NF‐κB is a nuclear transcription factor, named based on its specific binding with the enhancer κb sequence of κ light chain gene of B cell immunoglobulin. NF‐κB has been shown to play a role in drug resistance in malignant cancers such as glioma,^26^ breast cancer,^27^ myeloma,^28^ ovarian cancer,^29^ and melanoma.^30^ The efficacy of TMZ primarily depends on bioactive MTIC, which plays a cytotoxic role by alkylating O6 and N7 in guanine residues in DNA.^31^, ^32^ MGMT is an important downstream signaling molecule for NF‐κB participation in drug resistance of malignant tumor cells. It combines with the alkyl compounds at O6 in guanine residues in DNA, transfers the alkyl group to Cys‐145 at its active site, reduces the alkylated guanine, and prevents the appearance of a gap in the DNA sub chain, which confers drug resistance.^22^, ^33^, ^34^, ^35^ MGMT is the only protein that can remove the O6 guanine complex from DNA. Therefore, MGMT expression or activity in glioma cells may directly affect the resistance of cells to TMZ. Our study revealed that TMZ‐resistant glioma cells were characterized by high expression levels of RIP2 and MGMT, as well as enhanced NF‐κB activity. Conversely, the exogenous overexpression of RIP2 can induce activation of the NF‐κB pathway and upregulation of MGMT expression and also reduce sensitivity to TMZ. These results suggest that RIP2 may upregulate MGMT expression through NF‐κB in glioma cells and consequently confer resistance to TMZ.

JSH‐23 is an NF‐κB inhibitor that inhibits the nuclear translocation of NF‐κB p65 without affecting IκBα degradation.^36^ In vitro and in vivo studies have confirmed that JSH‐23 effectively inhibits NF‐κB transcription activity in a variety of tumor cells or tissues.^37^, ^38^, ^39^ Lomeguatrib is an MGMT inhibitor used for studying drug resistance in various tumors.^40^, ^41^ To further confirm the role of RIP2 in TMZ resistance, we constructed a drug‐resistant glioma cell xenograft model and treated it with TMZ after pretreatment with JSH‐23 or lomeguatrib. Pretreatment with JSH‐23 led to the downregulation of MGMT expression, while that with lomeguatrib did not exert the same effect; however, MGMT activity was inhibited in both cases. Notably, both JSH‐23 and lomeguatrib enhanced TMZ sensitivity in the cells, which consequently led to a pronounced antitumor effect. NF‐κB or MGMT could serve as potential targets in the treatment of RIP2‐positive TMZ‐resistant glioma.

In conclusion, our study revealed that RIP2 was upregulated in TMZ‐resistant glioma cells in correlation to drug resistance. The RIP2/NF‐κB/MGMT signaling pathway plays a vital role in the regulation of TMZ resistance. Therefore, combined treatment with an NF‐κB/MGMT inhibitor and TMZ enhances the therapeutic efficacy of the latter in RIP2‐positive TMZ‐resistant glioma.

## CONFLICT OF INTEREST

The authors do not have any possible conflicts of interest.

## Supporting information

Supplementary Material

## Data Availability

The data that support the findings of this study are available in the Supplementary Material of this article.
