# Supplementary material for: Regulation of temozolomide resistance in glioma cells via the RIP2/NF‐κB/MGMT pathway
Source: CNS Neurosci Ther. 2021 Jan 18;27(5):552–63. doi: 10.1111/cns.13591 (PMC8025621; doi:10.1111/cns.13591)
Supplement: Supplementary file 1 — Supplementary Material [file CNS-27-552-s001.pdf]

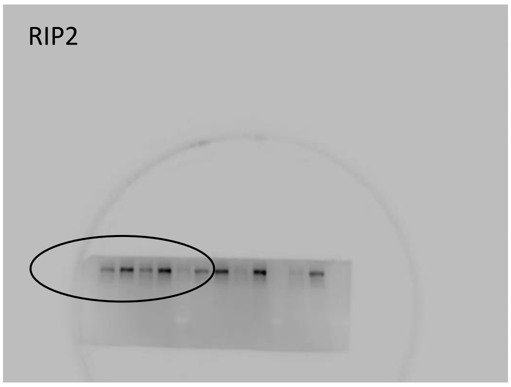

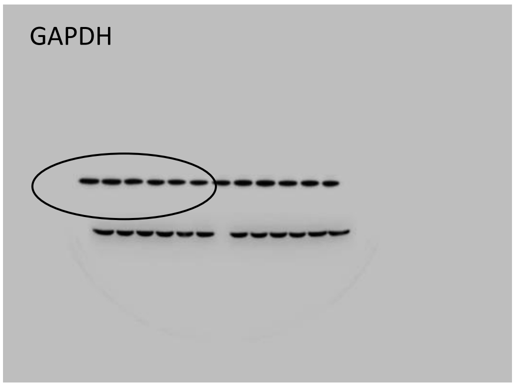

Full unedited gel/blot for Figure 1B

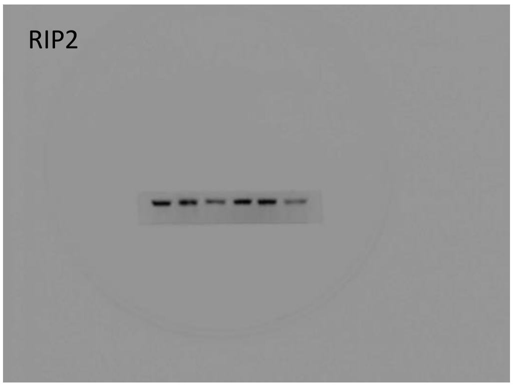

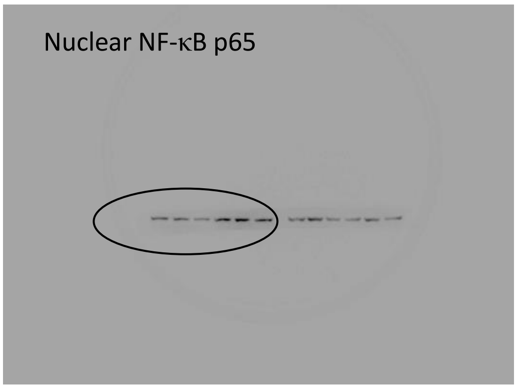

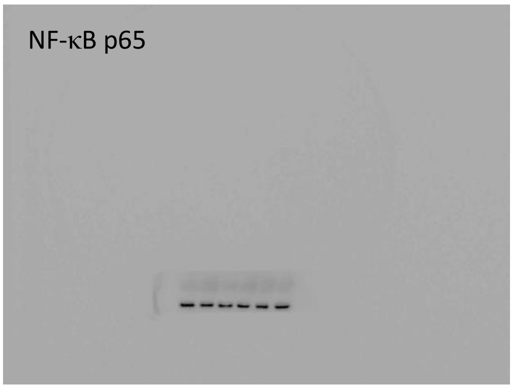

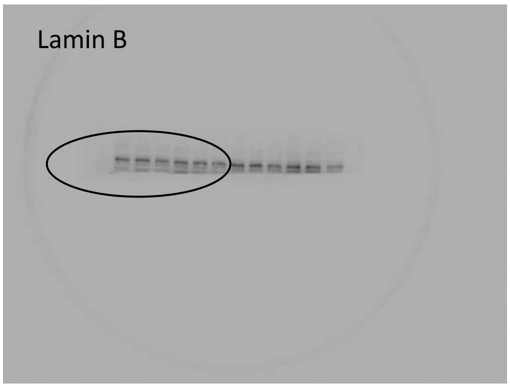

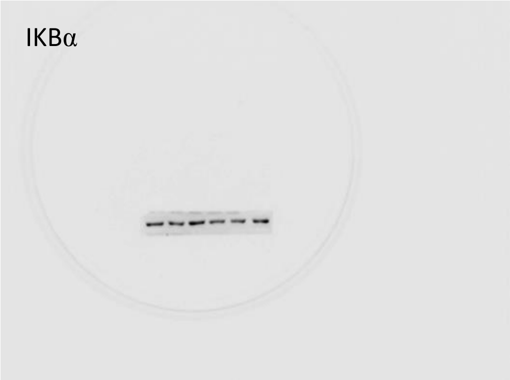

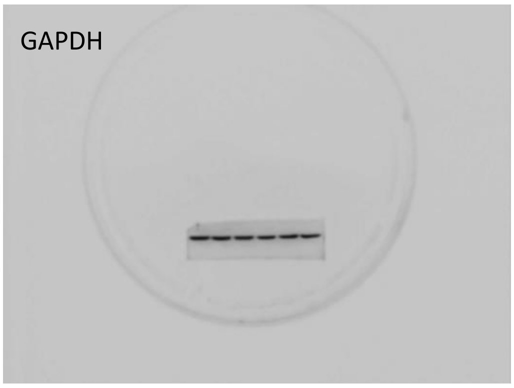

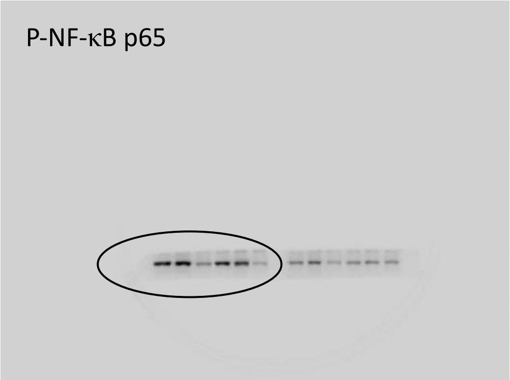

Full unedited gel/blot for Figure 2A

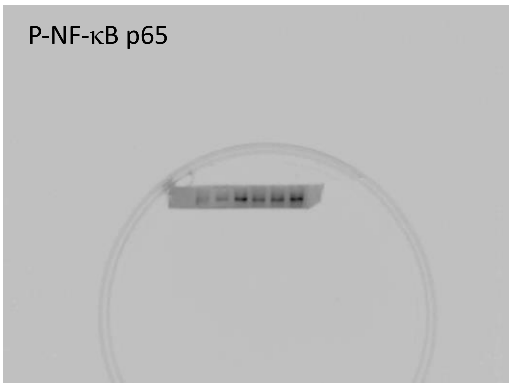

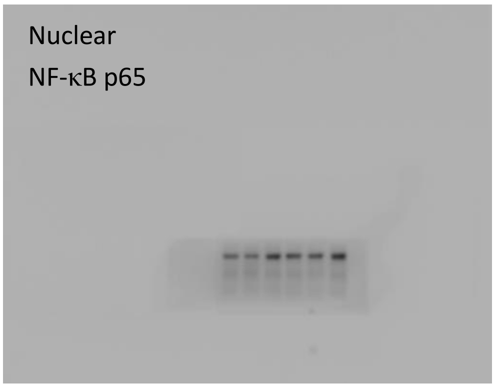

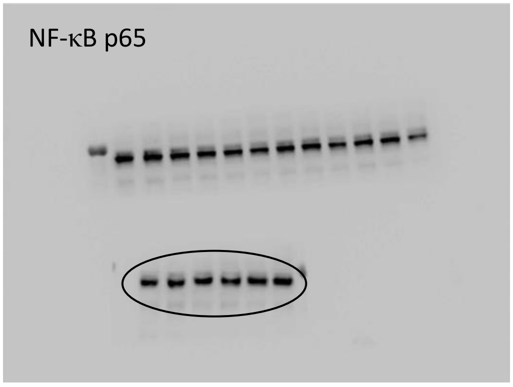

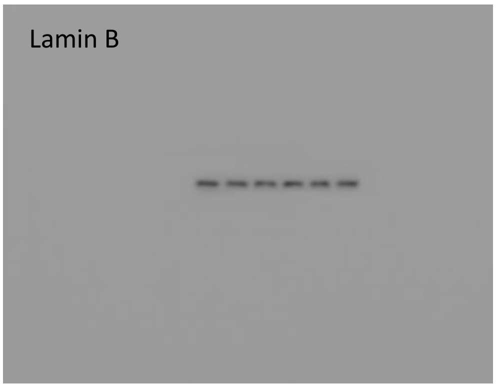

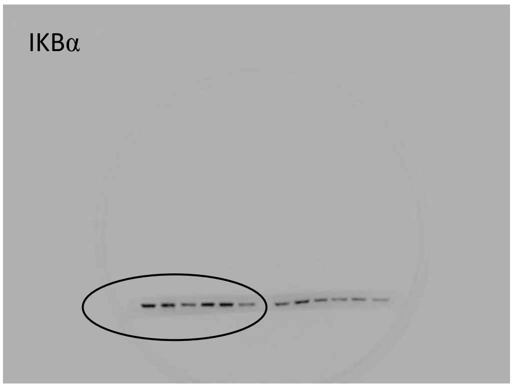

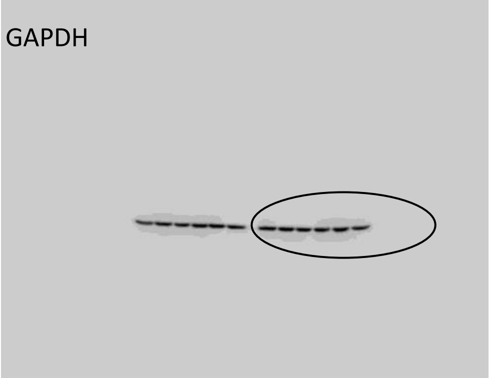

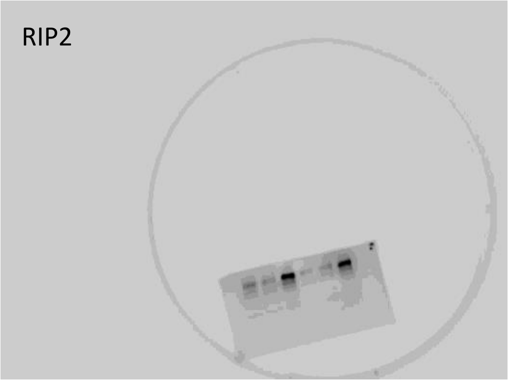

Full unedited gel/blot for Figure 2B

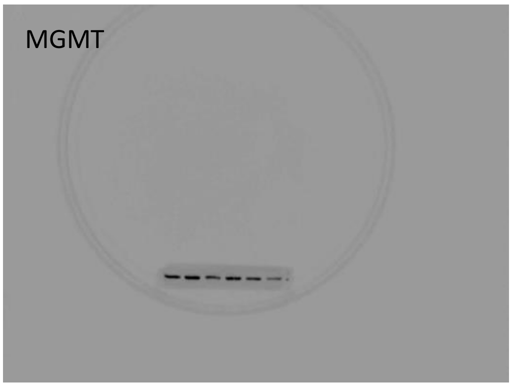

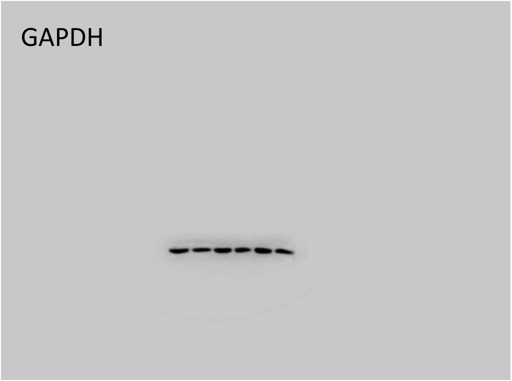

Full unedited gel/blot for Figure 3A

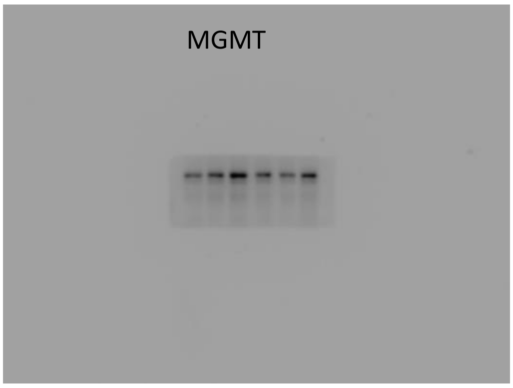

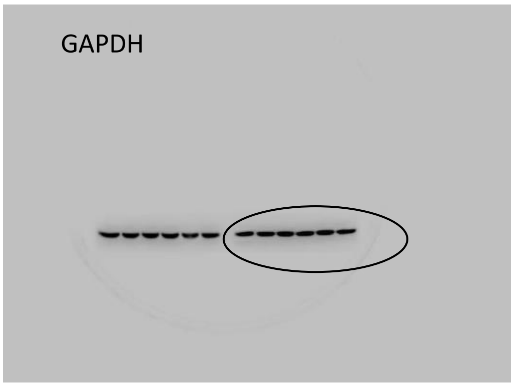

Full unedited gel/blot for Figure 3B

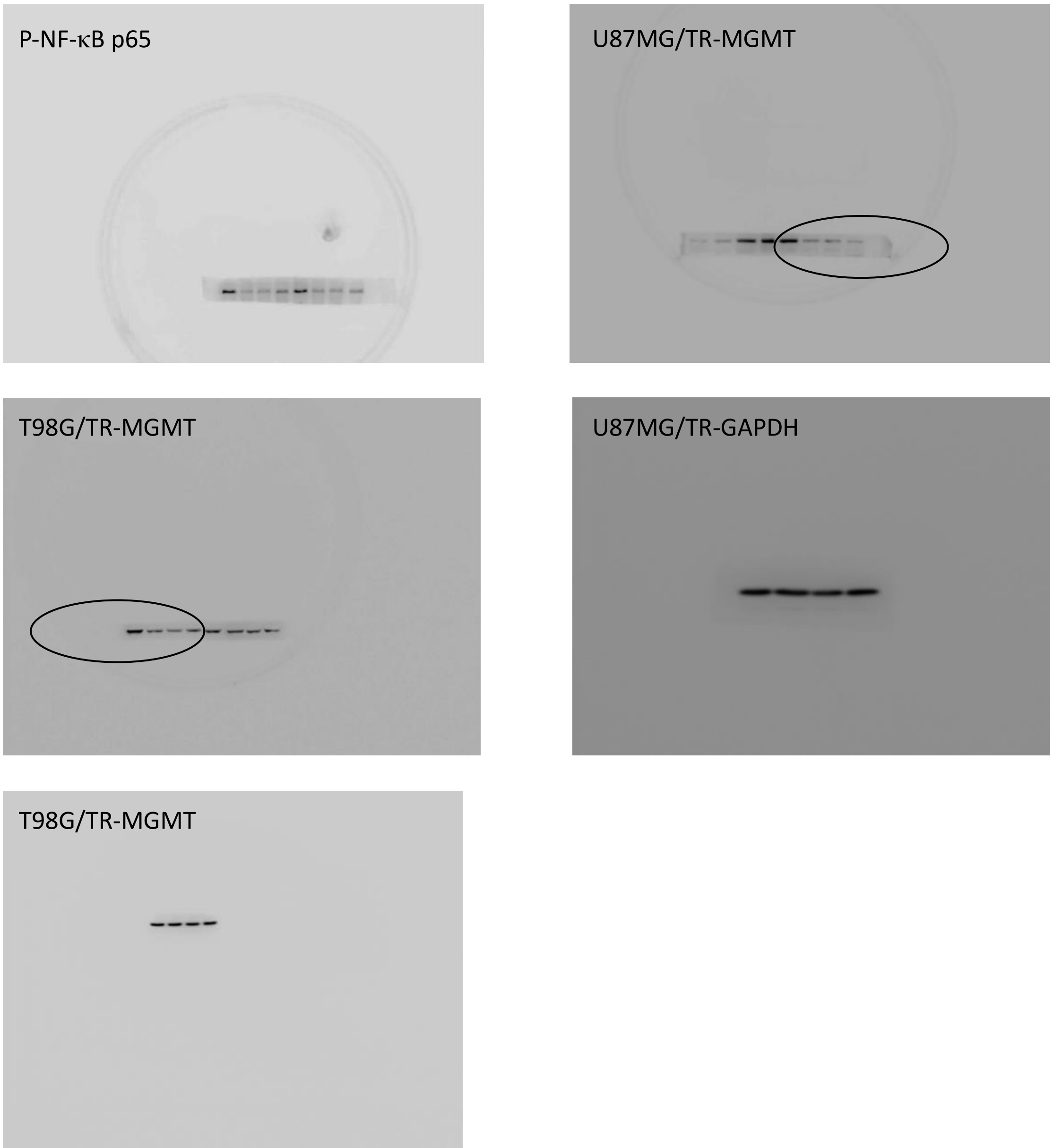

Full unedited gel/blot for Figure 4A

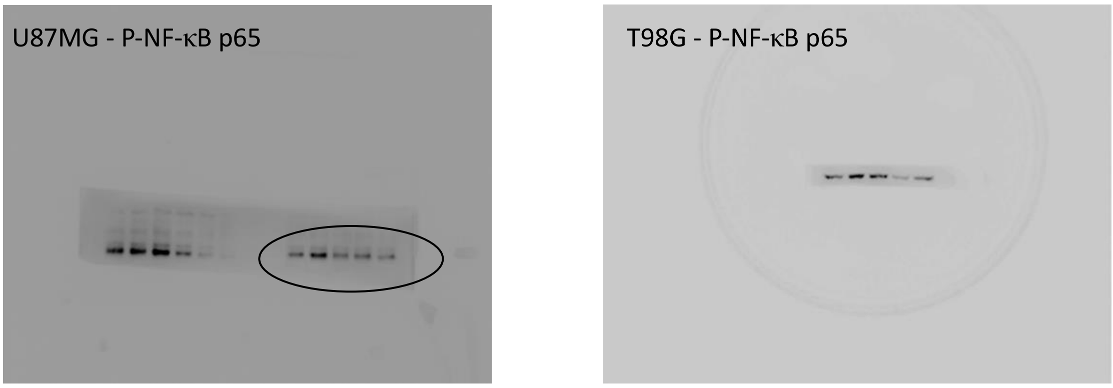

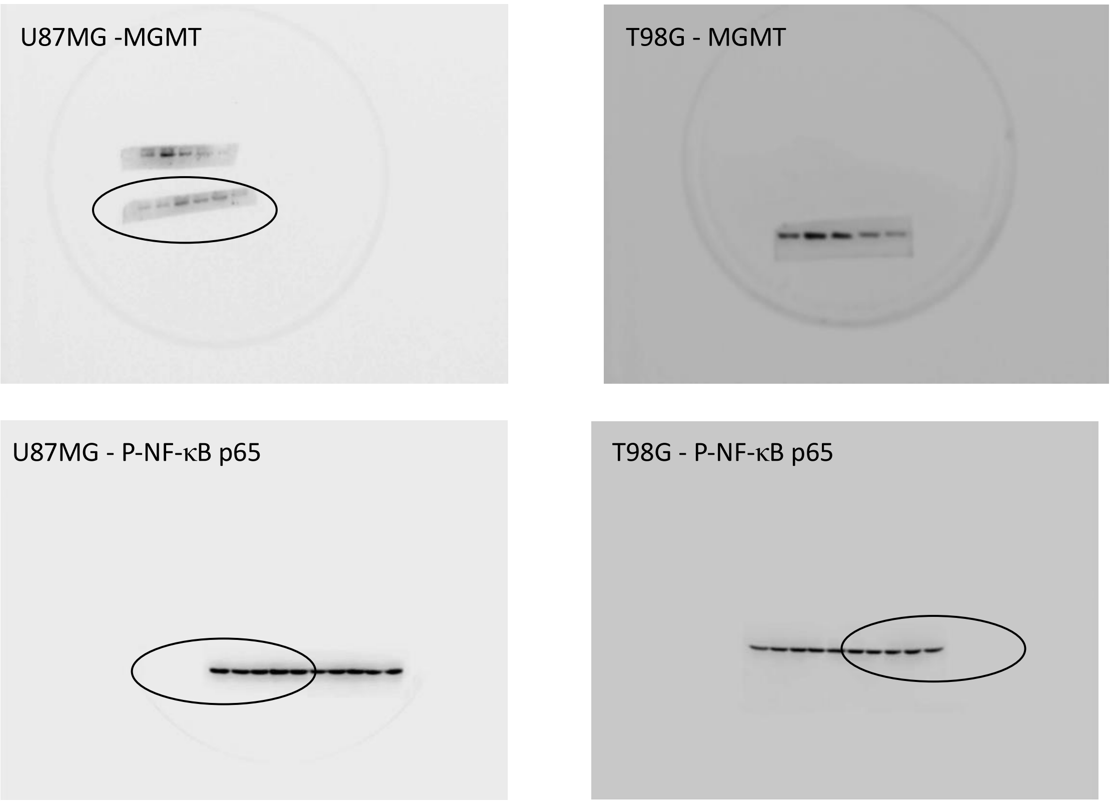

Full unedited gel/blot for Figure 4B

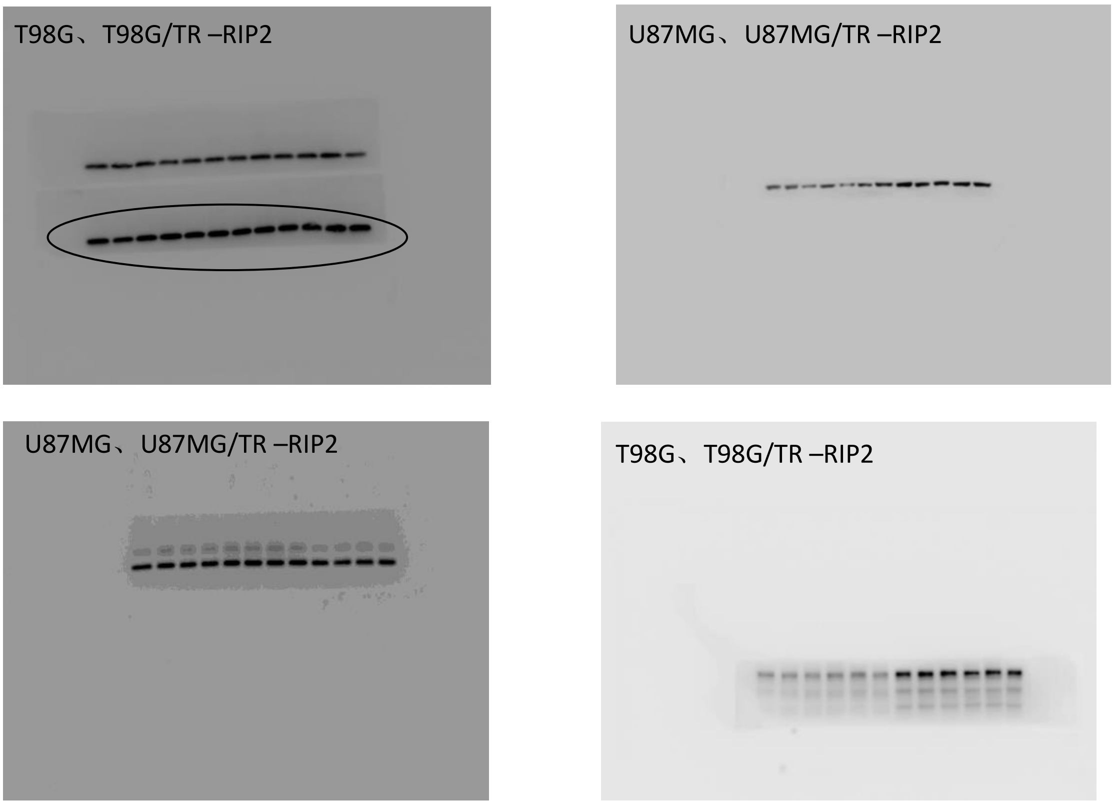

Full unedited gel/blot for Figure 6A
